# Associations of biomarkers with hypoattenuated leaflet thickening after transcatheter aortic valve replacement

**DOI:** 10.1016/j.rpth.2025.103341

**Published:** 2025-12-31

**Authors:** David Hesselbarth, Michelle D’Orazio, Giovanni Ciccarone, Diona Gjermeni, Carina Jülch, Philipp Breitbart, Philipp Ruile, Manuel Hein, Marius Wessinger, Mariya Maslarska, Jonathan Rilinger, Christopher Schlett, Fabian Bamberg, Klaus Kaier, Daniel Duerschmied, Torben Pottgiesser, Constantin von zur Mühlen, Dirk Westermann, Christoph B. Olivier

**Affiliations:** 1 Department of Cardiology and Angiology, University Heart Center Freiburg-Bad Krozingen, Faculty of Medicine, University of Freiburg, Freiburg, Germany; 2 Department of Diagnostic and Interventional Radiology, Medical Center, Faculty of Medicine, University of Freiburg, Freiburg, Germany; 3 Institute of Medical Biometry and Statistics, Faculty of Medicine and Medical Center, University of Freiburg, Freiburg, Germany; 4 Department of Cardiology, Angiology, Haemostaseology and Medical Intensive Care, University Medical Center Mannheim, Medical Faculty Mannheim, Heidelberg University, Heidelberg, Germany; 5 European Center for AngioScience, German Center for Cardiovascular Research partner site Heidelberg/Mannheim, Mannheim, Germany

**Keywords:** hypoattenuated leaflet thickening, HALT, valve thrombosis, hypercoagulability, inflammation, TAVR, transcatheter aortic valve replacement

## Abstract

**Background:**

Patients after transcatheter aortic valve replacement (TAVR) have a high incidence of hypoattenuating leaflet thickening (HALT). However, data on biomarkers related to HALT remain limited.

**Objectives:**

To identify hemostatic markers associated with HALT following TAVR.

**Methods:**

This prospective single-center cohort study assessed hemostatic profiles of patients undergoing TAVR between November 2020 and June 2022 using thrombelastography, light transmission aggregometry, and conventional laboratory markers. The primary outcome was moderate-to-severe HALT (grade 3/4) or reduced leaflet motion (grade 2/3) detected by computed tomography angiography at 6 months post-TAVR.

**Results:**

Of the 107 patients included, 68 had interpretable computed tomography angiography at 6 months. The primary outcome occurred in 14 (20%) patients. Biomarkers were measured at a median of 4 days (IQR, 3-6) after TAVR. Shortened clot formation time (R-time with heparin neutralization) indicated an association with HALT at 6 months (odds ratio [OR], 1.12 [95% CI, 1.00; 1.25]; *P* = .05) and demonstrated moderate discriminatory ability, with an optimal cutoff of 4.9 minutes (sensitivity 79%; specificity 74%; area under the curve, 0.74 [95% CI, 0.60; 0.89]; *P* = .005). Increased platelet count (OR, 1.08 [95% CI, 1.01; 1.15]; *P* = .03), elevated protein S (OR, 1.21 [95% CI, 1.01; 1.44]; *P* = .04), and higher C-reactive protein levels (OR, 1.19 [95% CI, 1.00; 1.41]; *P* = .05) also demonstrated associations with HALT. However, after adjustment for multiple testing using the Benjamini–Hochberg procedure, none of these associations was statistically significant.

**Conclusion:**

This exploratory study identified associations between HALT and biomarkers of hypercoagulability and inflammation, which did not persist after correction for multiple testing. These findings should be regarded as hypothesis-generating and warrant confirmation in future studies.

## Essentials

• HALT is frequent after TAVR.
• This prospective study used biomarkers and computed tomography to assess HALT after TAVR.
• Selected hemostasis and inflammation markers indicated possible associations with HALT.
• Findings need further study to clarify the roles of hemostasis and inflammation in HALT.

## Visual Abstract

**Figure:**
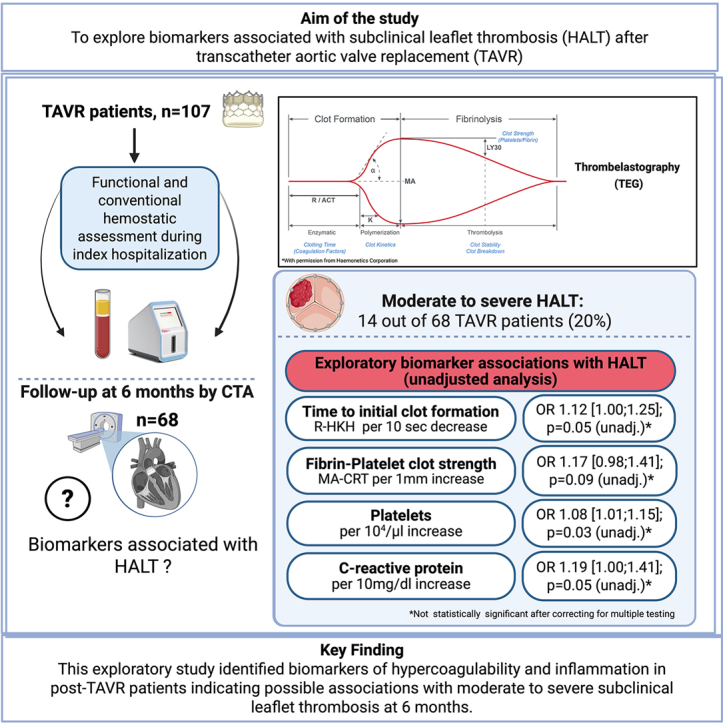

## 1 Introduction

Transcatheter aortic valve replacement (TAVR) for severe aortic stenosis can lead to early leaflet thrombosis, which may be asymptomatic or symptomatic, raising questions about optimal antithrombotic therapy in these patients [[1], [2], [3]]. Computed tomography angiography (CTA) is the reference standard for diagnosing leaflet thrombosis, which is typically identified as hypoattenuating leaflet thickening (HALT) or reduced leaflet motion (RLM). Previous clinical studies have detected HALT in up to one-third of asymptomatic patients following TAVR [1,4].

The clinical significance of HALT is still debated. While symptomatic hemodynamic valve deterioration is unlikely within the first year after TAVR in HALT patients [1,5], it may develop during extended follow-up [6], with potential implications for lifetime management strategies, particularly in younger patients. Some studies indicate an increased risk of stroke/transient ischemic attack [4,7] or mortality [8] in patients with HALT, whereas others do not observe a consistent association with clinical outcomes [5]. Given the high rates of HALT and the potential clinical impact on patients after TAVR, identifying markers for risk stratification is crucial.

While several studies have identified morphological and clinical risk factors [1,9,10], data on hemostatic markers for HALT prediction remain limited [[11], [12], [13]]. Conventional coagulation tests are limited by their *in vitro* nature and do not fully capture the *in vivo* coagulation system. Functional coagulation tests, such as light transmission aggregometry (LTA) and thrombelastography (TEG), have shown suggestive results in predicting cardiovascular events [14,15]. This study aimed to analyze a broad panel of biomarkers, with a focus on functional hemostatic markers, to inform risk stratification and explore their associations with HALT in TAVR patients.

## 2 Methods

### 2.1 Study design and cohort

This was an observational, prospective single-center cohort study. Patients were enrolled after TAVR, and hemostatic profiles were evaluated once during the index hospitalization (days 1-17 post-TAVR; median day, 4 [IQR, 3-6]). TAVR was performed according to state-of-the-art procedures. The primary outcome was defined as moderate-to-severe HALT (grade 3 or 4) or moderate-to-severe RLM (grade 2 or 3) and was assessed by CTA at 6 months. HALT was graded according to the Valve Academic Research Consortium (VARC)-3 criteria using a 5-point scale from the leaflet base to the free edge in the curvilinear dimension as follows: grade 0, no identifiable HALT; grade 1, HALT ≤25%; grade 2, HALT 26% to 50%; grade 3, HALT 51% to 75%; and grade 4, HALT >75%. Leaflet function was graded from 0 to 3 to define RLM in the curvilinear dimension from leaflet base to free edge as follows: grade 0, unrestricted leaflet motion; grade 1, mild restricted motion (<50%, limited to the leaflet base); grade 2, moderate restricted motion (involving the base and up to 50%-75% of the leaflet length); grade 3, severe restricted motion (>75%) [16]. When several leaflets had HALT or RLM, the leaflet with the highest grade was considered for the analysis.

This study (NCT03649594) was registered in a public trial database, conducted in accordance with the Helsinki Declaration, and approved by the Albert-Ludwigs-University Freiburg ethics committee. Patients undergoing TAVR were enrolled during hospitalization, excluding those with severe renal insufficiency (glomerular filtration rate < 30 mL/min/1.73 m^2^), severe thrombocytopenia (<50 × 10^3^/μL), or those undergoing Valve-in-Valve TAVR. Detailed inclusion and exclusion criteria are outlined in Supplementary Table S1. Patients were treated with single antiplatelet therapy (SAPT), dual antiplatelet therapy (DAPT), or anticoagulation as per standard of care.

### 2.2 Functional hemostatic assessment and blood samples

Venous blood was collected with a 21-gauge butterfly needle for standard laboratory tests and functional hemostatic assessment. A comprehensive list of the assessed hemostatic and inflammatory parameters is provided in Supplementary Table S4.

#### 2.2.1 TEG

TEG was performed using the TEG 6s hemostasis analyzer (Haemonetics Corporation), a 4-channel cartridge system that measures whole-blood clot viscoelasticity from the enzymatic to the fibrinolytic phase via thrombin pathway activation. To assess hemostasis, 1 cartridge includes 4 assays: citrated kaolin, citrated kaolin with heparinase, citrated rapid TEG, and citrated functional fibrinogen, as previously described [17]. Platelet function was assessed in a separate cartridge by selectively activating the adenosine diphosphate (ADP) and thromboxane pathways with ADP and arachidonic acid (AA), respectively. Overall hemostasis was assessed using kaolin activation and heparinase neutralization (HKH) of a heparinized blood sample. Fibrin’s contribution to clot formation, independent of platelets, was evaluated in the activator F channel using reptilase and factor XIII. TEG was assessed within 2 hours of the platelet cartridge and within 4 hours of the global hemostasis cartridge after blood sampling, following standard manufacturing protocols.

The R-time measures the duration in minutes until clot formation occurs. The α-angle, recorded in degrees, reflects the speed and kinetics of clot solidification. Maximum amplitude (MA) measures the maximum clot strength in millimeters, reflecting the combined contribution of cross-linked fibrin and activated platelets. In contrast, MA for citrated functional fibrinogen specifically represents the strength of the fibrin mesh without the platelet contribution. The Ly30 value, expressed as a percentage, indicates the proportion of the clot that has dissolved 30 minutes after reaching MA. Representative curves are presented in Supplementary Figure S4.

#### 2.2.2 LTA

LTA was performed using the PAP-8E Platelet Aggregometer (Bio/Data Corporation) according to the manufacturer’s standard protocol (mölab point-of-care).

After optical calibration of each channel using distilled water, a blank was set using 225 μL platelet-poor plasma combined with 25 μL distilled water. Siliconized test tubes containing 225 μL platelet-rich plasma were incubated under continuous stirring at 37 °C. After incubation, 25 μL aliquots were added to each tube to achieve the following final reagent concentrations: AA, 1.64 mM; thrombin receptor-activating peptide, 10 μM and 5 μM; ADP, 2 μM and 20 μM. Light transmission (%) was plotted continuously for 10 minutes after the start of the assay and is proportional to platelet aggregation over time. Key LTA parameters included primary aggregation, MA, and disaggregation induced by AA or ADP. Representative curves are presented in Supplementary Figure S5.

### 2.3 Post-TAVR-CTA protocol

CTA of the aortic root was performed on a second- or third-generation dual-source scanner (Somatom Definition Flash or Somatom Definition Force, Siemens Healthineers) using retrospective electrocardiogram gating in a cranio-caudal direction. A total of 50 mL of iodinated contrast agent (Imeron 400, Bracco, or Ultravist 370, Bayer Healthcare) was injected at a flow rate of 4 mL/s, followed by 40 mL of isotonic saline solution at the same rate. A CTA scan was initiated using a bolus-tracking technique, with the region of interest placed in the left atrium. All CTA images were reconstructed at 50-millisecond steps throughout the cardiac cycle, with a slice thickness of 1 mm, an increment of 0.8 mm, and a stent-specific reconstruction kernel (B46f).

### 2.4 Outcomes and follow-up

The primary study outcome was defined as the presence of at least 1 prosthetic valve leaflet with HALT grade 3 or 4 or RLM grade 2 or 3 (moderate-to-severe), identified on CTA at 6 months. Three experienced readers (M.H., P.R., and P.B., all certified at the highest level in Cardiac-CTA by the German Cardiac Society) supervised the HALT analysis independently and resolved discrepancies by consensus. Follow-up was performed at 6 months ± 12 weeks, or earlier if clinically indicated.

### 2.5 Statistical analysis

The sample size calculation was based on preliminary TEG data from percutanous coronary intervention patients, indicating a higher thrombin-induced MA in those who experienced ischemic events [14]. We assumed a similar association with subclinical leaflet thrombosis after TAVR, which occurs in 15% of patients [9]. To detect a 5 mm difference in thrombin-induced MA between patients with and without leaflet thrombosis, 92 participants (14 with thrombosis and 78 without) were required to achieve 80% power, with a type I error rate of .05. Accounting for a 10% dropout rate, a total of 103 participants were needed.

Categorical variables are presented as numbers and frequencies, and continuous variables as medians and IQR. Categorical variables were compared using Fisher’s exact test. Logistic regression was used to analyze predictors of HALT, and pressure gradients were compared using the Kruskal–Wallis test and the Mann–Whitney *U*-test. Two-tailed tests with *p* values ≤ .05 were considered significant. Data were analyzed using Prism 10.2.0 (GraphPad) and SPSS 29.0.0.1 (IBM).

## 3 Results

### 3.1 Patient population

**Figure 1:**
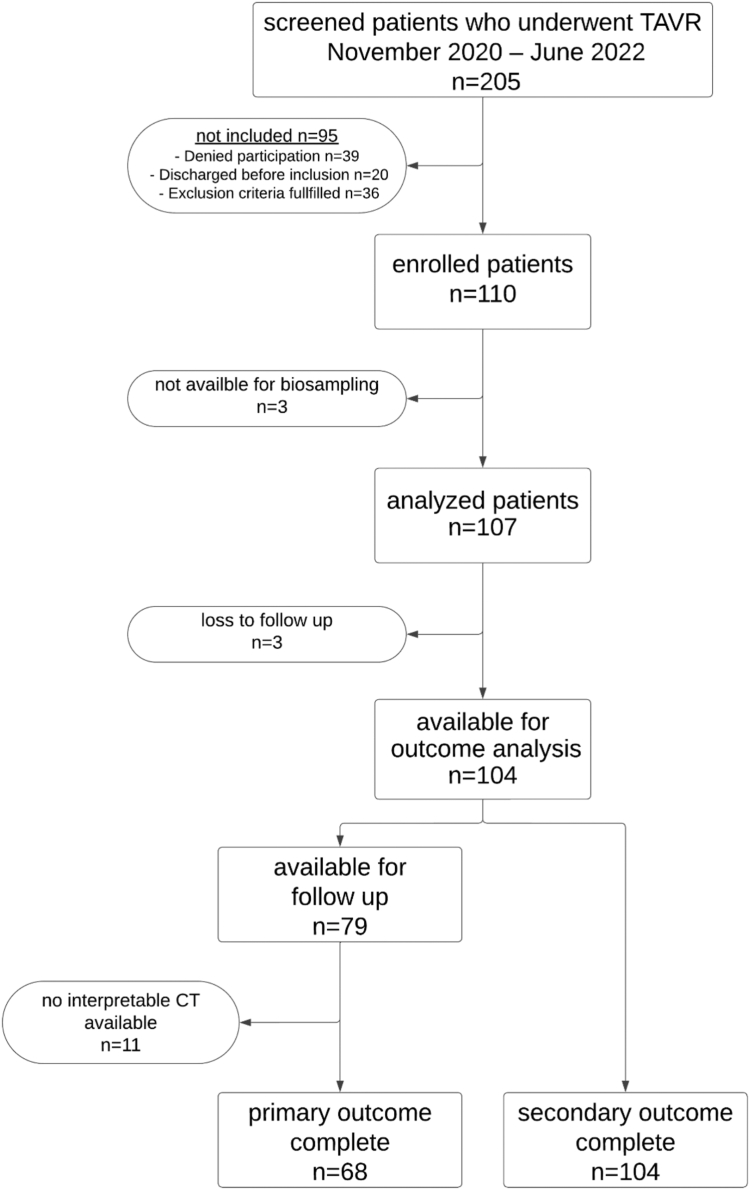
Flow chart of patient selection. CT, computed tomography; TAVR, transcatheter aortic valve replacement.

Between November 2020 and June 2022, 205 patients were screened, and 107 were enrolled in this single-center, prospective, observational cohort study within 17 days post-TAVR (median day, 4 [IQR, 3-6]; Figure 1). Clinical follow-up was completed for 104 patients, and interpretable CTA was available for 68. Baseline characteristics are provided in Table 1. The median age was 82 years (IQR, 79-85), with 55 males (51%). Balloon-expandable valves were used in 93 patients (87%), while 14 (13%) received self-expanding valves. At discharge, 27 patients (25%) were on SAPT, 33 (31%) on DAPT, and 47 (44%) on oral anticoagulation (OAC) with or without antiplatelet therapy (OAC ± APT). Detailed antithrombotic therapy at discharge and follow-up is provided in Supplementary Table S2. The median (IQR) time to functional hemostatic measurement was 33 minutes (IQR, 30; 40) for TEG and 75 minutes (IQR, 40; 100) for LTA, with the measured parameters detailed in Supplementary Table S4.

**Table 1:** Clinical baseline characteristics.

Baseline characteristics	Total (*N* = 107)
Demography	
Age (y)	82 (79-85)
Male	55 (51)
BMI (kg/m^2^)	25.1 (23.3-27.3)
Patient medical history	
CHA_2_DS_2_-VASc score	5 (4-6)
HAS-BLED	3 (2-4)
Atrial fibrillation	56 (52)
TIA/stroke	21 (20)
Myocardial infarction	12 (11)
History of bleeding	13 (12)
History of VTE	10 (9)
Coronary artery disease	76 (71)
PAD	9 (8)
Chronic kidney failure	32 (30)
History of cancer	22 (21)
Arterial hypertension	103 (96)
Diabetes mellitus	25 (23)
Smoking	16 (15)
Positive family history	11 (10)
Procedural characteristics	
Balloon-expandable valve	93 (87)
Self-expanding valve	14 (13)
Antithrombotic therapy at discharge	
SAPT	27 (25)
DAPT	33 (31)
OAC ± APT	47 (44)

Values are expressed as median (IQR) or *n* (%).

APT, antiplatelet therapy; BMI, body mass index; CHA_2_DS_2_VASC score, congestive heart failure, hypertension, age ≥ 75, diabetes mellitus, stroke/transient ischemic attack, vascular disease, age 65-74, sex category; DAPT, dual antiplatelet therapy; HAS-BLED, hypertension, abnormal renal and liver function, stroke, bleeding history or predisposition, labile international normalized ratio, elderly, drugs or alcohol; OAC, oral anticoagulation; PAD, peripheral artery disease; SAPT, single antiplatelet therapy; TIA, transient ischemic attack; VTE, venous thromboembolism.

### 3.2 Hemostatic markers as predictors of HALT

At 6 months, 68 of 107 patients had interpretable CTAs, with any HALT in 29 patients (29/68; 43%). The primary outcome, defined as the presence of at least 1 prosthetic valve leaflet with HALT grade 3 or 4 or RLM grade 2 or 3 (moderate-to-severe), occurred in 14 patients (14/68; 20%).

**Figure 2:**
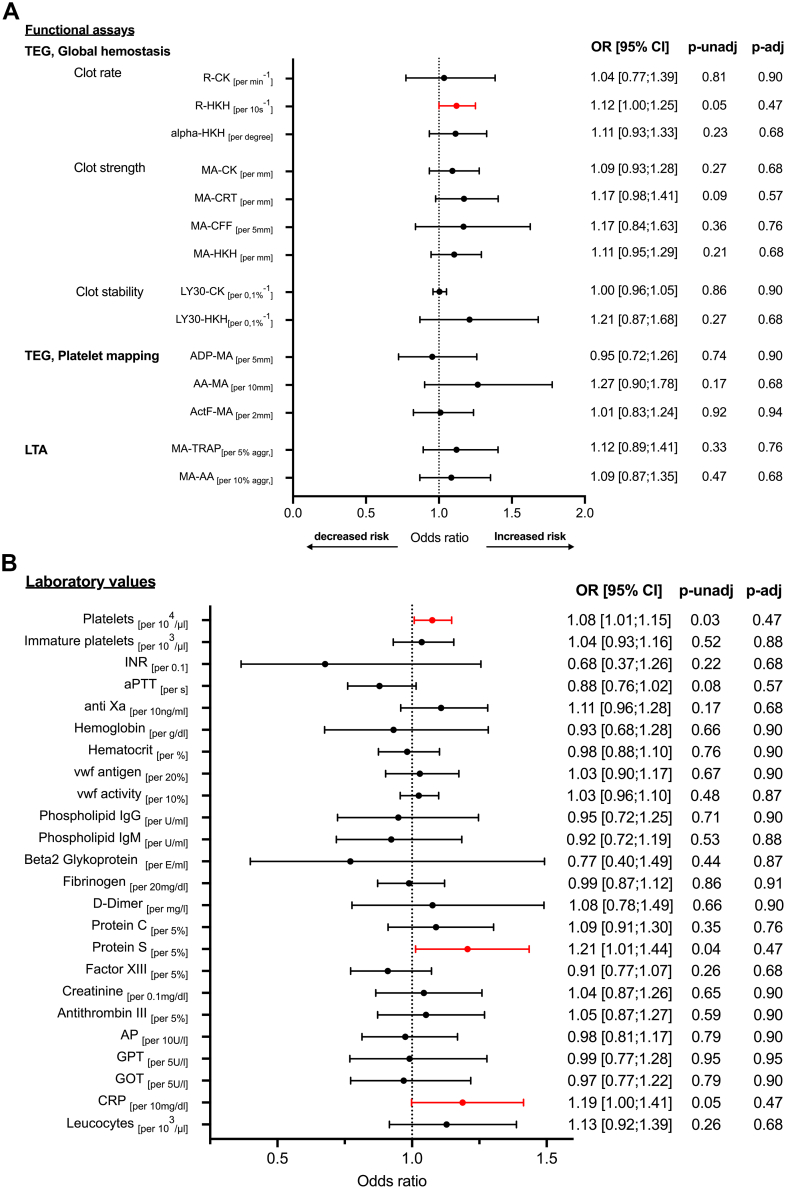
(A and B) Biomarkers associated with the occurrence of hypoattenuating leaflet thickening grade 3 or 4 or reduced leaflet motion grade 2 or 3 at 6 months. Logistic regression with functional assays (A) and laboratory values (B) measured after transcatheter aortic valve replacement during index hospitalization. Hypoattenuating leaflet thickening was diagnosed with a computed tomography angiography scan 6 months posttranscatheter aortic valve replacement. Increased risk indicates hypercoagulability. Decreased risk indicates hypocagulabilty. AA, arachidonic acid; ActF, activator F; ADP, adenosine diphosphate; aggr., aggregation; alpha, kinetics of clot formation; AP, alkaline phosphatase; aPTT, activated partial thromboplastin time; CFF, citrated functional fibrinogen; CK, citrated kaolin; CRP, C-reactive protein; CRT, citrated rapid TEG; GOT, gulatamate oxaloacetate transaminase; GPT, glutamate pyruvate transaminase; HKH, heparinized kaolin with heparinase; Ig, immunoglobulin; INR, international normalized ratio; LTA, light transmission aggregometry; Ly30, thrombolysis 30 minutes after reaching MA; MA, maximum aggregation; OR, odds ratio; p-adj, adjusted *P* value; p-unadj, unadjusted *P* value; R, time to initial clot formation; s, second; TEG, thrombelastography; TRAP, thrombin receptor-activating peptide; VWF, von Willebrand factor.

Functional hemostatic assessment using TEG global hemostasis indices, platelet mapping, and LTA—specifically R-time (clot initiation), MA (clot strength), and Ly30 (fibrinolysis)—demonstrated numerical associations with the primary outcome across different agonists, without reaching statistical significance (Figure 2A).

Among the evaluated functional hemostatic parameters, TEG R-time with HKH (R-HKH) demonstrated an association with moderate-to-severe HALT or RLM at 6 months prior to correction for multiple testing. Shorter R-HKH was associated with higher odds of the primary outcome (odds ratio [OR], 1.12 [95% CI, 1.00; 1.25] per 10-second decrease; *P* = .05; Figure 2A) and demonstrated moderate discriminatory ability, with an optimal cutoff of 4.9 minutes (sensitivity 79%; specificity 74%; area under the curve, 0.74 [95% CI, 0.60; 0.89]; *P* = .005). After adjustment for discharge regimen (SAPT, DAPT, and OAC), this association was attenuated and no longer statistically significant (OR, 1.14 [95% CI, 0.97-1.34]; *P* = .11 per 10-second decrease).

Among the other assessed functional hemostatic parameters, overall platelet-fibrin clot stability (MA) and AA-induced MA on both TEG and LTA were numerically higher in patients with moderate-to-severe HALT or RLM (per 1-mm increase in MA-citrated rapid TEG: OR, 1.17 [95% CI, 0.98-1.41], *P* = .09; per 10-mm increase in AA-MA on TEG: OR, 1.27 [95% CI, 0.90-1.78], *P* = .17; per 10% increase in AA-MA on LTA: OR, 1.09 [95% CI, 0.87-1.35], *P* = .47), whereas clot lysis (Ly30) was numerically lower (per 0.1% decrease in Ly30-HKH: OR, 1.21 [95% CI, 0.87-1.68]; *P* = .27). After correction for multiple testing, none of the functional hemostatic parameters, including R-HKH, was statistically significant.

Representative TEG curves (Supplementary Figure S4A, B) illustrate a shorter R-HKH and a higher MA-HKH in a patient with HALT compared with a patient without HALT. Representative LTA curves (Supplementary Figure S5A, B) similarly demonstrate a higher MA activated by thrombin receptor-activating peptide or AA in the patient with HALT. Individual TEG and LTA measurements for the corresponding parameters are provided in Supplementary Figure S6.

For soluble hemostatic markers from standard laboratory testing, associations with the primary outcome were observed for platelet count and protein S levels (per 10^4^/μL increase in platelets: OR, 1.08 [95% CI, 1.01-1.15], *P* = .03; per 5% increase in protein S: OR, 1.21 [95% CI, 1.01-1.44], *P* = .04; Figure 2B). Numerical associations without statistical significance were observed for activated partial thromboplastin time and international normalized ratio (per second increase in activated partial thromboplastin time: OR, 0.88 [95% CI, 0.76; 1.02], *p* = .08; per 0.1 increase in the international normalized ratio: OR, 0.68 [95% CI, 0.37; 1.26]; *p* = .22).

Regarding inflammatory markers, increased C-reactive protein (CRP) levels demonstrated an association with moderate-to-severe HALT or RLM (per 10 mg/dL increase in CRP: OR, 1.19 [95% CI, 1.00; 1.41]; *P* = .05), while leukocyte counts showed a numerical, nonsignificant association (per 10^3^/μL increase: OR, 1.13 [95% CI, 0.92-1.39]; *P* = .26). After correction for multiple testing, none of the soluble hemostatic or inflammatory markers remained statistically significant (Figure 2B). Time from blood draw was associated with an increase in platelets (14.8 × 10^3^/μL [95% CI, 9.7; 19.9] per day after TAVR; *P* < .001), whereas no associations were observed for CRP, R-HKH, protein S, or moderate-to-severe HALT (Supplementary Figure S3A, B).

### 3.3 Clinical and procedural characteristics associated with HALT

Major adverse cardiovascular events (MACE) occurred in 9 of 104 patients (9%) who completed clinical follow-up, and clinically relevant bleeding events (VARC types 1-3) were observed in 10 (10%) patients (Table 2). Of the 14 patients with moderate-to-severe HALT or RLM, 1 patient experienced clinically relevant bleeding, and none experienced MACE during the 6-month follow-up period. Atrial fibrillation was associated with a lower risk of developing moderate-to-severe HALT or RLM (OR, 0.12 [95% CI, 0.03; 0.61]; *P* = .01). In patients with HALT, 2 of 14 (14%) were on OAC ± APT, while 12 of 14 (86%) received APT alone. In the APT group only, 9 were on DAPT, whereas 3 received SAPT. The difference across regimens was statistically significant (*P* = .01). Coronary artery disease was associated with HALT (OR, 9.65 [95% CI, 1.18; 79.10]; *P* = .03; Supplementary Figure S1).

**Table 2:** Secondary outcomes.

Secondary outcome	Total (*N* = 104)
MACE	9 (9)
Death	4 (4)
Myocardial infarction	1 (1)
TIA/stroke	5 (5)
Bleeding events	25 (24)
VARC type 1	4 (4)
VARC type 2	2 (2)
VARC type 3	4 (4)
Not classified in VARC	15 (14)

Values are expressed as *n* (%).

MACE, major adverse cardiovascular event; TIA, transient ischemic attack; VARC, Valve Academic Research Consortium.

After TAVR, 32 (31%) patients were hospitalized during the 6 ± 2-month follow-up period, of whom 14 (14%) were hospitalized for procedures or cardiovascular events (Supplementary Table S3). Patients with moderate-to-severe HALT or RLM did not have a higher likelihood of hospitalization or cardiovascular/procedure-related hospitalization (hazard ratio, 1.23 [95% CI, 0.35; 4.30], *P* = .74; hazard ratio, 1.60 [95% CI, 0.19; 13.29], *P* = .66, respectively). The median mean pressure gradients by echocardiography at the time of CTA were numerically higher in patients with moderate-to-severe HALT or RLM compared with those without, though the difference was not statistically significant (11.90 mmHg [95% CI, 8.50; 14.10] vs 10.10 mmHg [95% CI, 7.75; 13.18]; *P* = .25; Supplementary Figure S2A, B).

## 4 Discussion

In this exploratory study, we evaluated whether a broad panel of blood biomarkers, including functional hemostatic measures, was associated with the occurrence of moderate-to-severe HALT or RLM. The main findings are: (1) At 6 months, HALT was detected in 43% of TAVR patients, and moderate-to-severe HALT or RLM in 20%. (2) Early postprocedural biomarkers, including clot formation time, platelet count, protein S, and CRP, demonstrated associations with moderate-to-severe HALT, but none was statistically significant after multiple testing correction.

The HALT rate in this study was higher than in other studies, likely due to differences in the timing of HALT assessment and in the HALT criteria [4]. When assessed at 6 months or later using the VARC-3 criteria, the rates were only slightly higher than those reported in other studies [10,18].

While anticoagulant therapy appears effective in preventing HALT [11], it can still occur in patients receiving anticoagulation. In this study, 2 of 14 patients (14%) developed moderate-to-severe HALT despite anticoagulation treatment. Consistent with other studies, DAPT did not protect against HALT [4], with 9 of 14 patients (57%) still affected. The clinical outcome of those with HALT is controversial, making the benefit of anticoagulation unclear [[4], [5], [6],^8^,^18^]. While current evidence does not support routine anticoagulation in post-TAVR patients [2,19], no randomized trial has systematically evaluated the potential benefits of intensified antithrombotic therapy in those with HALT. Histological analysis of TAVR prostheses with HALT showed thrombus formation within 30 days, whereas later stages exhibited more organized thrombi, suggesting that early anticoagulation may be more effective [3]. Of note, our study was not designed to assess the efficacy of antithrombotic therapy and should not inform therapeutic strategies. Analogous to Virchow’s triad, proposed mechanisms for HALT include altered flow dynamics in the aortic neosinus, endothelial damage, and hypercoagulability [20]. Several studies have identified morphological and clinical risk factors [1,9,10], but there is limited data on hemostatic markers as potential indicators of hypercoagulability.

The novelty of this study lies in its comprehensive analysis of biomarkers, including functional measurements with TEG and LTA, in patients prospectively enrolled after TAVR and screened for HALT 6 months later. Patients with moderate-to-severe HALT or RLM at 6-month follow-up exhibited early postprocedural differences in several hypercoagulability- and inflammation-related biomarkers during hospitalization after TAVR. These included associations with shorter clot formation time, higher platelet counts, elevated protein S, and increased CRP levels, as well as numerical trends toward greater maximal clot stability and reduced fibrinolysis on TEG. These findings should be interpreted with caution, as all associations were observed only in the unadjusted analysis and none remained statistically significant after correction for multiple testing.

The observed increase in protein S levels in patients with moderate-to-severe HALT or RLM is unexpected, given that reduced protein S is typically associated with prothrombotic states. Possible explanations include a compensatory response to hypercoagulability; acute-phase dynamics, as protein S can be influenced by inflammatory mediators and correlates with CRP [21]; or analytical factors, such as hypertriglyceridemia or hyperbilirubinemia, which may artifactually raise protein S antigen measurements [22]. Time to initial clot formation (ie, R-time) was associated with the primary outcome only when heparin was neutralized (ie, R-HKH). This finding could not be significantly detected by conventional hemostatic analysis (Figure 2A, B). Limited data from other studies have shown conflicting results. While some studies found no significant association between coagulation markers and HALT [11], a recent study reported that pre-TAVR von Willebrand factor activity, decreased post-TAVR hemoglobin, and increased post-TAVR lactate dehydrogenase were associated with HALT at 3 months post-TAVR [12]. In our study, we assessed hemostatic markers post-TAVR and conducted CTA scans at 6 months, which may partially explain the differing findings.

Elevated CRP levels showed an association with HALT, whereas leukocyte counts demonstrated only numerical differences. This aligns with a prior retrospective analysis of 385 patients, in which CRP—but not leukocyte counts—was associated with HALT when measured before TAVR [13]. Histopathological studies of prosthetic leaflets with HALT have reported inflammatory cell infiltration, suggesting that inflammatory processes may be involved, although causality remains unproven [3].

Given the number of statistical tests performed, the likelihood of type I error increases; after false discovery rate adjustment (Benjamini–Hochberg), none of the associations remained statistically significant. Additionally, patients with available CTA constituted a healthier subset than those without CTA (Supplementary Table S5), limiting generalizability. Nevertheless, the observed directional trends in several TEG parameters and platelet-related markers indicate that potential contributions from both plasmatic and platelet pathways cannot be excluded. However, given the small number of events and the exploratory nature of the analysis, these findings should be interpreted cautiously and do not allow for a firm conclusion.

Interpretation of post-TAVR biomarkers is challenging, as the acute-phase response can influence measured levels. HALT likely reflects a multifactorial process involving flow dynamics, device-related factors, and blood-material interactions. In this context, our biomarker assessment provides exploratory, nondefinitive signals that early hypercoagulability and inflammation could be associated with HALT development. However, given the exploratory design, the limited number of events, and the lack of statistically significant associations after correction, these observations should be interpreted cautiously. Larger studies are needed to clarify these potential relationships.

At the 6-month follow-up, patients with moderate-to-severe HALT or RLM did not exhibit a higher rate of MACE or clinically significant bleeding compared with those without. However, bias may exist if event-affected patients missed follow-up CTA scans (Supplementary Table S6). Moreover, the occurrence of MACE in HALT patients has been reported in previous studies, with conflicting results [4,5,8].

Transthoracic echocardiography revealed slightly higher mean pressure gradients in patients with moderate-to-severe HALT or RLM, though the difference was not statistically significant (Supplementary Figure S2A, B). Similarly, studies assessing pressure gradients at comparable time points post-TAVR found no significant differences [1,18]. However, long-term follow-up may reveal valve deterioration in patients with HALT [6,7], underscoring its relevance to long-term prognosis.

### 4.1 Strengths and limitations of the study

This is the first study to evaluate a comprehensive panel of biomarkers, including functional tests, and assess their association with HALT in post-TAVR patients. The strength of this study lies in its assessment of functional hemostatic markers, providing additional data beyond conventional laboratory tests. The main limitation is the small sample size, which reduces the statistical power of the findings. Blood samples were collected near discharge, but the single time-point measurement and variable post-TAVR clinical conditions may have influenced the results and precluded assessment of temporal change during hospitalization. Additionally, the absence of follow-up CTA may have introduced bias, as healthier patients were more likely to attend follow-up.

## 5 Conclusion

Selected biomarkers of hypercoagulability and inflammation measured after TAVR were associated with moderate-to-severe HALT or RLM of the valve prosthesis on CTA at 6 months follow-up. However, these associations were not significant after correction for multiple testing and should therefore be considered hypothesis-generating. Larger, adequately powered studies are needed to determine whether these biomarkers play a role in elucidating HALT pathophysiology or to inform future risk stratification and study design.
