# Supplementary material for: Associations of biomarkers with hypoattenuated leaflet thickening after transcatheter aortic valve replacement
Source: Res Pract Thromb Haemost. 2025 Dec 31;10(1):103341. doi: 10.1016/j.rpth.2025.103341 (PMC12861211; doi:10.1016/j.rpth.2025.103341)
Supplement: Supplementary Material [file mmc1.docx]

**Supplemental**

**Supplemental Table S1: Inclusion and exclusion criteria**

| **Inclusion criteria** | - Informed consent - ≥18 years of age |
| --- | --- |
| **Exclusion criteria** | - GFR (MDRD) <30ml/min/173m² - Previously known coagulopathy - Any other condition as determined by the PI. (e.g. incompliance, drug abuse, unwillingness to adhere to the protocol) - Valve-in-Valve transcateter aortic valve replacement |

**Table S2. Clinical Baseline Characteristics**

| Baseline characteristics | Total n=107 |
| --- | --- |
| Procedural characteristics | |
| Edwards Sapien 3  20mm  23mm  26mm  29mm | 93 (87%)  4 (4%)  32 (30%)  41 (38%)  16 (15%) |
| Medtronic Evolut R  23mm  26mm  29mm  34mm | 14 (13%)  1 (1%)  4 (4%)  8 (8%)  1 (1%) |
| Antithrombotic therapy at discharge | |
| ASA mono therapy  DAPT  Clopidogrel mono | 24 (22%)  33 (31%)  3 (3%) |
| ASA + OAC  DAPT + OAC  P2Y12 Inhibitor + OAC  OAC mono | 25 (23%)  12 (11%)  4 (4%)  6 (6%) |
| Antithrombotic therapy at follow up | |
| ASA mono therapy  DAPT  Clopidogrel | 32 (30%)  16 (15%)  1 (1%) |
| ASA + OAC  P2Y12 Inhibitor + OAC  OAC mono | 3 (3%)  9 (8%)  43 (40%) |
| Not Known | 3 (3%) |
| Values expressed in median (Interquartile range [IQR]) or n (%).  Abbreviations: ASA, acetylsalicylic acid; DAPT, Dual antiplatelet therapy; OAC, Oral anticoagulation | |

**Table S3. Secondary outcomes**

| *Secondary outcome* | Total n=104 |
| --- | --- |
| Hospitalized patients  Reason for rehospitalization  Procedure/valve related  Other cardiovascular  Heart failure  Non-cardiovascular  Hospitalized > 1 time | 32 (31%)  1 (1%)  13 (13%)  5 (5%)  18 (17%)  5 (5%) |
| Values expressed in n (%). | |

**Table S4. Hemostatic parameters**

| Functional hemostatic parameters | Median (IQR) |
| --- | --- |
| TEG, Global hemostasis |  |
| R-CK [min] | 7.4 (6.7-8.3) |
| R-CKH [min] | 7.2 (6.5-8.3) |
| R-HKH [min] | 5.2 (4.35-5.8) |
| alpha-CRT [degree] | 78.5 (77.1-79.85) |
| MA-CK [mm] | 65.3 (62.25-66.9) |
| MA-CKH [mm] | 65 (61.95-68.25) |
| MA-HKH [mm] | 65.8 (63.9-68.7) |
| MA-CRT [mm] | 66.6 (64.2-69.2) |
| MA-CFF [mm] | 30.9 (25.05-37.8) |
| a10-CFF [mm] | 28.8 (24.2-35) |
| LY30-CK [%] | 0.7 (0.1-1.5) |
| LY30-CRT [%] | 0.1 (0-0.4) |
| TEG, Platelet mapping |  |
| AA-MA [mm] | 35 (20.3-53.55) |
| ADP-MA [mm] | 57.7 (53-62.35) |
| ActF-MA [mm] | 19.9 (17.6-23.2) |
| LTA |  |
| MA-TRAP [%] | 80 (72-90) |
| MA-AA [%] | 20.5 (13-51.5) |
| FA-AA [%] | 17.5 (10-48.5) |
| DA-AA [%] | 0 (0-4) |
| PA-AA [%] | 20.5 (13-51.5) |
| FA-ADP [%] | 63.5 (38-75.25) |
| MA-ADP [%] | 68 (56-79) |
| DA-ADP [%] | 5 (0-16) |
| PA-ADP [%] | 68 (56-79) |
| **Laboratory parameters** |  |
| Platelets [tsd/µl] | 165 (131-227.5) |
| Immature platelets [tsd/µl] | 7.35 (5.425-9.875) |
| INR | 1.05 (1.005-1.12) |
| aPTT [s] | 31 (29-35) |
| Anti Xa [ng/ml] | 108 (53.25-187.5) |
| Hämoglobin [g/dl] | 10.7 (9.5-12.5) |
| Hämatokrit [%] | 32 (28.2-37.25) |
| Vwf antigen [%] | 204 (162-257) |
| Vwf activity [%] | 195 (147-265) |
| Phospholipid IgG [U/ml] | 4 (3-6) |
| Beta2 Glykoprotein [E/ml] | 2 (1-2) |
| Phospholipid IgM [U/ml] | 2 (2-4) |
| D-Dimer [mg/l] | 1.56 (0.94-2.255) |
| Fibrinogen [mg/dl] | 461 (413.5-538) |
| Protein C [%] | 90 (80-101.5) |
| Protein S [%] | 78 (68-92.9) |
| Faktor XIII [%] | 120 (104.5-136.25) |
| Antithrombin III [%] | 92 (82-103) |
| Creatinine [mg/dl] | 0.96 (0.785-1.17) |
| GPT [U/l] | 22 (15-30.5) |
| GOT [U/l] | 30 (23-39.5) |
| AP [U/l] | 79.5 (63-97.75) |
| Leucocytes [tsd/µl] | 8.01 (6.765-9.45) |
| CRP [mg/dl] | 32.3 (16.3-53.45) |
| Parameters expressed in median (Interquartile range, IQR). |  |

**Table S5. Baseline characteristics in the overall cohort and in patients with versus without CTA**

| *Baseline characteristics* | All patients  n=107 | CTA available  n=68 | CTA unavailable  Total n=39 |
| --- | --- | --- | --- |
| Demography | |  |  |
| Age [years] | 82 (79-85) | 80 (77-83) | 85 (82-87) |
| Male | 55 (51%) | 35 (51%) | 20 (51%) |
| BMI | 25.1 (23.3-27.3) | 25.1 (23.4-26.8) | 25.0 (22.2-27.6) |
| Patient medical history | |  |  |
| CHA_2_DS_2_-VASc score | 5 (4-6) | 5 (4-6) | 6 (5-6) |
| HAS-BLED | 3 (2-4) | 3 (2-3) | 3 (3-4) |
| Atrial fibrillation | 56 (52%) | 33 (49%) | 23 (59%) |
| TIA/stroke | 21 (20%) | 10 (15%) | 11 (28%) |
| Myocardial infarction | 12 (11%) | 9 (13%) | 3 (8%) |
| History of bleeding | 13 (12%) | 6 (9%) | 7 (18%) |
| History of VTE | 10 (9%) | 5 (7%) | 4 (10%) |
| Coronary artery disease | 76 (71%) | 44 (65%) | 32 (82%) |
| PAD | 9 (8%) | 5 (7%) | 4 (10%) |
| Chronic kidney failure | 32 (30%) | 15 (22%) | 17 (44%) |
| History of cancer | 22 (21%) | 12 (18%) | 10 (26%) |
| Arterial hypertension | 103 (96%) | 65 (96%) | 38 (97%) |
| Diabetes mellitus | 25 (23%) | 19 (28%) | 6 (15%) |
| Smoking | 16 (15%) | 11 (16%) | 5 (13%) |
| Positive family history | 11 (10%) | 10 (15%) | 1 (3%) |
| Procedural characteristics | |  |  |
| Balloon expandable valve | 93 (87%) | 56 (82%) | 37 (95%) |
| Self-expanding valve | 14 (13%) | 12 (18%) | 2 (5%) |
| Antithrombotic therapy at discharge | |  |  |
| SAPT | 27 (25%) | 15 (22%) | 10 (26%) |
| DAPT | 33 (31%) | 21 (31%) | 12 (31%) |
| OAC +/- APT | 47 (44%) | 32 (47%) | 17 (44%) |
| **Secondary outcome** |  |  |  |
| MACE  Death  Myocardial infarction  TIA/stroke | 9 (9%)  4 (4%)  1 (1%)  5 (5%) | 1 (1%)  0 (0%)  0 (0%)  1 (1%) | 8 (21%)  4 (10%)  1 (3%)  4 (10%) |
| Bleeding events  VARC Type 1  VARC Type 2  VARC Type 3  not classified in VARC | 25 (24%)  4 (4%)  2 (2%)  4 (4%)  15 (14%) | 19 (28%)  4 (6%)  0 (0%)  2 (3%)  13 (19%) | 6 (15%)  0 (0%)  2 (5%)  2 (5%)  2 (5%) |

| Values expressed in median (Interquartile range [IQR]) or n (%).  Abbreviations: BMI, Body-Mass-Index; TIA, Transient ischaemic attack; VTE, Venous thromboembolism; PAD, Peripheral artery disease; SAPT, Single antiplatelet therapy; DAPT, Dual antiplatelet therapy; OAC, Oral anticoagulation; APT, Antiplatelet therapy, HALT, Hypoattenuated Leaflet Thickening; Mace, Major adverse cardiovascular events; TIA, Transient ischaemic attack; VARC, Valve Academic Research Consortium, CTA: Computed tomography angiography |
| --- |


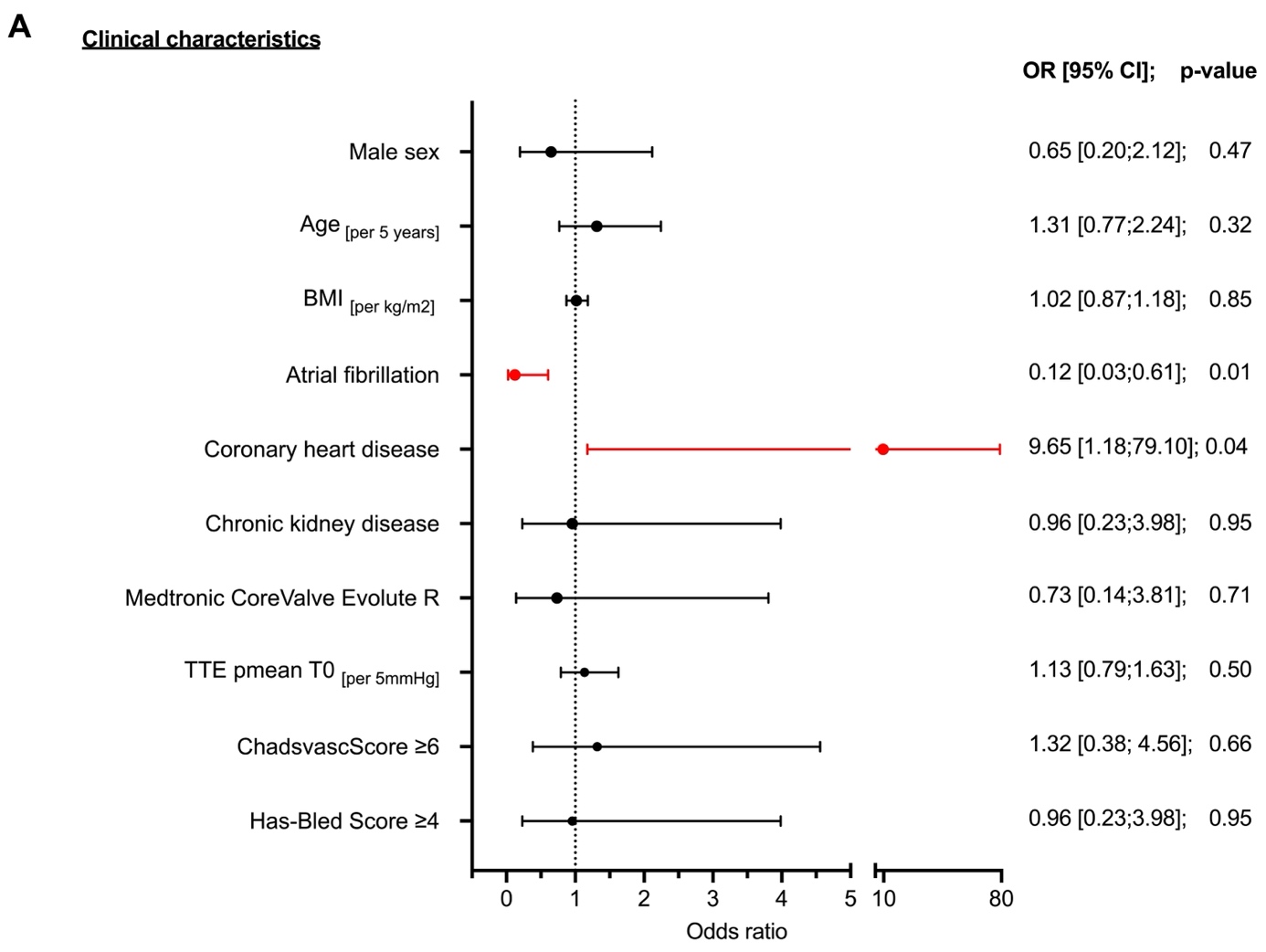


**Figure S1.** Logistic regression with clinical characteristics associated with the occurrence of HALT within 6 months. B: COX regression with clinical characteristics as predictors for the occurrence of MACE within 6 months C: COX regression with clinical characteristics as predictors for the occurrence of bleeding within 6 months. MACE: Major adverse cardiac event, HR: Hazard ratio, BMI: Body mass index, TTE: Transthoracic echocardiography. T0: measurement during hospitalization

**Figure S2 A+B.** Mean pressure gradients in transthoracic echocardiography at 6 months. HALT: Hypoattanuated leaflet thickening. RLM: Reduced leaflet motion


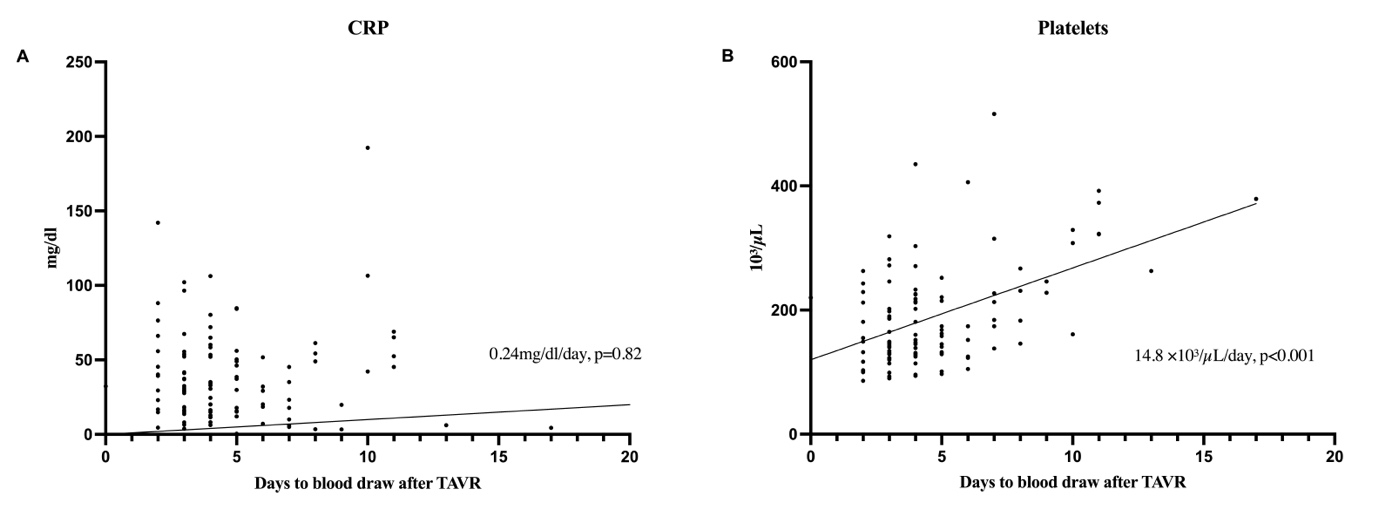


***Figure S3 A+B*** **Association of CRP and platelets with timing of blood draw after TAVR**. (A) CRP levels by days from TAVR to blood draw showing no significant association. (B) Platelet counts increased significantly from time to blood draw.

**A**

**B**

**Figure S4 A+B.** TEG curves in patients without HALT (A) and with HALT (B). The TEG curves from the patient with HALT shows a shorter R-HKH and higher MA-HKH compared with the patient without HALT.

**A**


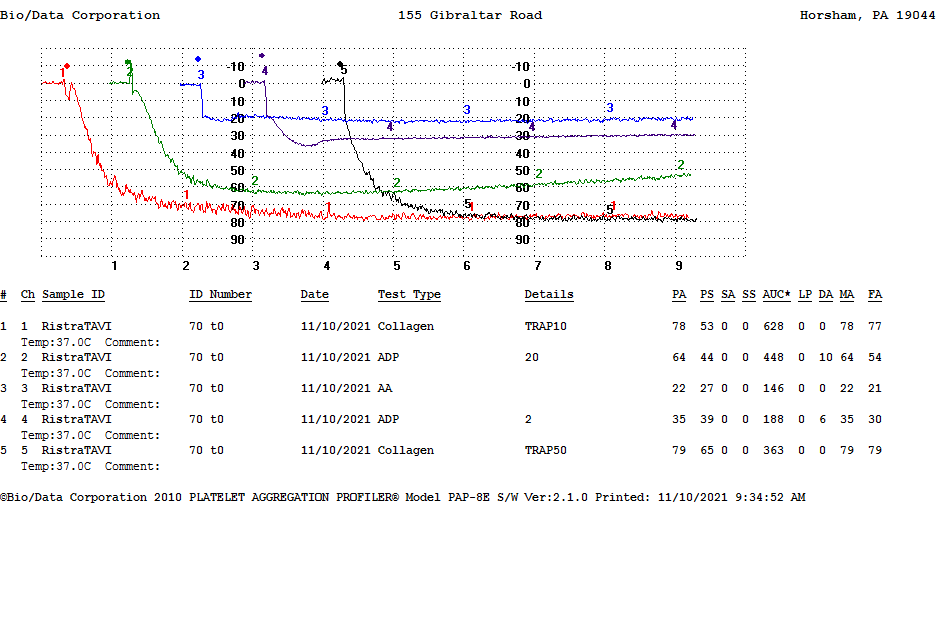


**B**


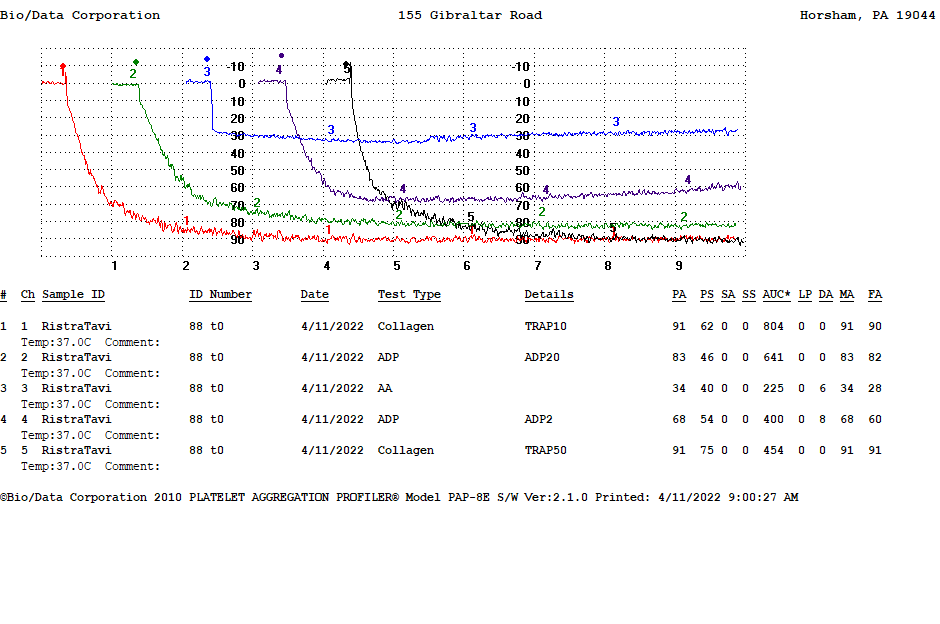


**Figure S5 A+B**: LTA curves in patients without HALT (A) and with HALT (B). The LTA curve from the patient with HALT demonstrates higher MA-TRAP and MA-AA compared with the patient without HALT.


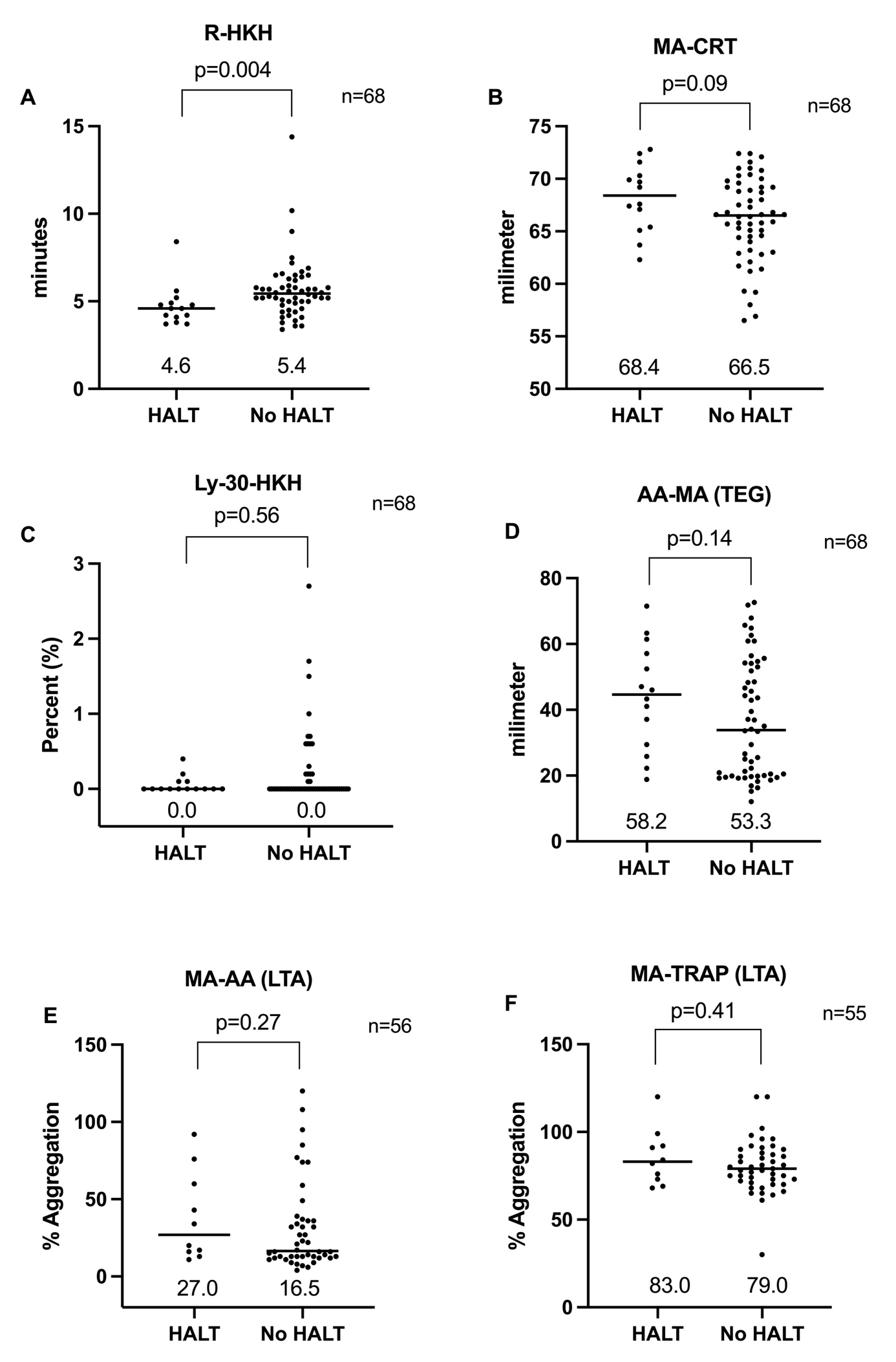


**Figure S6 (A–F).** Individual patient data for functional hemostatic parameters measured at a median of 4 days (IQR 3–6) after TAVR. Median values are indicated numerically. HALT refers to patients with moderate to severe hypoattenuated leaflet thickening; No HALT refers to patients without moderate to severe HALT. Group comparisons were performed using the Mann–Whitney U test.TEG: thrombelastography; LTA: light transmission aggregometry; R-HKH: time to initial clot formation; MA: maximum amplitude; Ly30: clot lysis 30 minutes after MA; HKH: heparinized kaolin with heparinase; AA: arachidonic acid
